# Women’s Satisfaction with Body Image before Pregnancy and Body Mass Index 4 Years after Delivery in the Mothers of Generation XXI

**DOI:** 10.1371/journal.pone.0070230

**Published:** 2013-07-31

**Authors:** Ana Henriques, Elisabete Alves, Henrique Barros, Ana Azevedo

**Affiliations:** 1 Department of Clinical Epidemiology, Predictive Medicine and Public Health, University of Porto Medical School, Porto, Portugal; 2 Institute of Public Health – University of Porto, Porto, Portugal; University of Bologna, Italy

## Abstract

**Background:**

Body image satisfaction (BIS) influences body weight regulation and may contribute to long-term healthier lifestyle after pregnancy. Thus, we aimed to assess the association between BIS before pregnancy and body mass index (BMI) 4 years after the index pregnancy.

**Methods:**

As part of the follow-up of a birth cohort, 3612 women with prepregnancy BMI >18.5 kg/m^2^ were reevaluated 4 years after the birth of a child. BIS was defined as the difference between perceived and ideal body size before pregnancy, assessed by Stunkard Silhouettes after birth. The associations of BIS with BMI change (continuous) and BMI classes at 4 years, based on measured weight and height, were estimated using linear and multinomial regression, respectively.

**Results:**

Among women with normal prepregnancy BMI, those who felt too small, regarding their ideal, had a 0.25 kg/m^2^ smaller increase in BMI within 4 years and a lower likelihood of becoming overweight or obese [multivariate-adjusted odds ratio (OR) = 0.63; 95% confidence interval (95%CI): 0.44–0.91 and OR = 0.21; 95%CI: 0.05–0.91, respectively) than those satisfied with body image. Feeling too large was associated with a 0.41 kg/m^2^ larger increase in BMI and a higher risk of becoming overweight or obese (OR = 2.12; 95%CI:1.73–2.59 and OR = 3.42; 95%CI:2.02–5.79, respectively). A similar, non-significant, trend was observed for overweight women. Obese women who felt too large had a non-significant decrease in BMI.

**Conclusions:**

BIS plays a role in maternal body weight after delivery. Realistic body size goals may promote the motivation to lose weight and contribute to higher success in attaining them.

## Introduction

Worldwide, obesity is rapidly increasing particularly at younger ages and more women of reproductive age present excess weight [1], [2]. In Portugal, the overall prevalence of overweight and obesity is also rising [3], [4], with 40% of women in their thirties being overweight or obese [5].

Women are particularly vulnerable to become overweight after pregnancy [6]. A substantial weight increase occurs at this stage of women’s reproductive life which may alter their weight change trajectory [7]. Currently, many women gain more weight than recommended during pregnancy [8], carrying an immediate higher risk of pregnancy and delivery complications [9], and gestational weight gain is associated with weight change postpartum [10], [11]. A higher prepregnancy body mass index (BMI), higher weight gain during pregnancy and more weight retaining after delivery all contribute to women becoming overweight after pregnancy [6].

There is clear evidence that obesity is linked with poor body image [12]–[16], particularly in women [17], [18] and body image could play a role in the social and psychological experience of being obese. Body image is a complex and multidimensional construct, containing both cognitive (attitudinal) and affective components [19]. It is essential to distinguish which specific dimension each study intends to address, namely considering the mechanisms involved and the potential for intervention, and to choose a tool that better measures that specific dimension [20]. In the present study, the attitudinal dimension will be assessed, specifically focusing on the evaluation component represented by self-ideal discrepancies to measure body satisfaction valuations [21].

Body image satisfaction (BIS) plays an important role in weight gain also during pregnancy [7]. Excessive weight gain during pregnancy and retention after delivery can be justified as common and unavoidable [8], and some women have a more relaxed attitude to weight gain in this period of time. This may reflect the feeling of less pressure to be thin after pregnancy, as the role of “mother” confers respectability to weight gains that would otherwise be unacceptable [22]. Conversely, some women reported a significant decline in body image satisfaction from prepregnancy to early pregnancy [23] and this variation is extended to the postpartum period [7], although the nature of these disparities between women is not clear. Mothers who feel more dissatisfied with their bodies immediately after pregnancy have significantly greater long term weight gains. However, understanding the effect of prepregnancy BIS on postpartum weight changes is expected to be more relevant and amenable to planned intervention included in preconception and prenatal care, strengthening spontaneous changes in BIS related with the experience of motherhood [22].

Thus, BIS may be useful to identify women who are more susceptible of gaining excessive weight earlier. Therefore, we tested the hypothesis of a possible association between BIS before pregnancy, using the difference of the measures of current and desired body size, and BMI 4 years after delivery in mothers of a Portuguese population-based birth cohort.

## Methods

### Ethics Statement

The study protocol was approved by the Ethics Committee of Hospital de São João and by the Portuguese Authority of Data Protection on 27^th^ of April, 2005 and was carried out in accordance with the principles of the 1964 Declaration of Helsinki. Written informed consent was obtained from all participants at baseline and follow-up evaluation. Were eligible to participate all women hospitalized for childbirth in one of the five public maternity units covering the metropolitan area of Porto, Portugal whose obstetric outcome was a live birth with at least 24 weeks of gestation. Of the invited mothers, 91.4% accepted to participate. All potential participants who declined to participate or otherwise did not participate were not disadvantaged in any other way by not participating in the study.

This study is based on the birth cohort Generation XXI which has been previously described [24]. Briefly, in 2005–2006, 8495 women, who gave birth to 8647 infants, were enrolled into the cohort after the child’s birth. The recruitment occurred in all five public maternity clinics that at the time covered the metropolitan area of Porto, Portugal. During the hospital stay and within 72 hours after delivery, trained interviewers using structured questionnaires were responsible for data collection on demographic and socioeconomic characteristics, lifestyles, medical history, anthropometrics and body image perception.

Four years later, in 2009–2011, a follow-up evaluation took place, and 84.2% of the mothers were reevaluated. Of all mothers, 67.4% attended a face-to-face interview and physical examination at the study site, 16.8% provided self-reported data by telephone interview, leaving 15.8% lost to follow-up. In this study, women interviewed by telephone were excluded due to the lack of anthropometric data.

For the current analysis, we excluded 313 participants who had been recruited and evaluated from the first trimester of pregnancy to address specific objectives [25], since body image perception is expected to change during pregnancy. We also excluded 131 mothers with BMI <18.5****kg/m^2^ at baseline because they were too few to be analyzed separately and probably very different, regarding BIS, from those with normal BMI to be aggregated in the same category; 164 mothers who were pregnant at the follow-up visit; and 1578 with missing data on any of the socio-economic characteristics, smoking before pregnancy, parity, weight gain during pregnancy, breastfeeding, BMI before pregnancy and 4 years after delivery, or BIS. Therefore, we included in the analyses 3612 women with prepregnancy BMI ≥18.5****kg/m^2,^ who participated in the re-evaluation of the cohort and had complete information for the key variables considered.

Overall, among all mothers in the baseline cohort, those with complete data to be included in the final sample for analysis were older (≥35 years: 18.2% vs. 15.1%, *p*<0.001) had a higher educational level (schooling >12 years: 26.3% vs. 22.2%, *p*<0.001) and higher household income (>1500/month: 29.3% vs. 26.8%, p<0.001). Regarding BIS before pregnancy, they were more likely to consider themselves too large regarding their ideal body image (49.9% vs. 42.2%, *p*<0.001) and they were also more frequently overweight/obese at baseline (BMI ≥25.0∶31.6% vs. 29.1%, *p*<0.001) than those who did not have all the information for the key variables.

### Baseline Evaluation

Age was categorized as less than 25 years, 25 to 29 years, 30 to 34 years and 35 years or above. Education was categorized as less than compulsory elementary schooling (9 years), secondary school (10 to 12 years) and higher education (>12 years). Household monthly income was inquired using previously defined categories: less than 1000 €, 1001 to 1500 €, more than 1500 € and women who didn’t know or preferred not to answer.

Parity was recorded as the number of deliveries for each participant at baseline, including the delivery of the infant included in the cohort Generation XXI.

Assessment of exposure to smoking included information on the daily number of cigarettes smoked before and during pregnancy. Current smokers included both daily and occasional smokers, and those who had previously smoked but not in the last 3 months before pregnancy were considered former smokers.

At birth, prepregnancy weight was self-reported to the nearest 0.1 Kg and, assuming that a young adult woman does not have considerable changes in her height over four years [26], we used the height measured at the follow-up to calculate prepregnancy BMI. Mother’s prepregnancy BMI was categorized according to the standard World Health Organization definition: underweight (<18.5 kg/m^2^), normal (18.5–24.9 kg/m^2^), overweight (25.0–29.9 kg/m^2^) and obese (≥30 kg/m^2^) [23]. Weight gain during pregnancy was considered as the difference between the mother’s reported weight immediately before delivery and prepregnancy weight, and was categorized as bellow, above or as recommended, according to the Institute of Medicine guidelines [27].

BIS before pregnancy was calculated as the difference between perceived self body size and perceived ideal body size, both assessed at the baseline evaluation, immediately after delivery, by the Stunkard Silhouettes [28]. This scale consists of 9 silhouette figures that increase gradually in size from very thin to very obese (1 to 9, respectively). BIS was categorized as women who were satisfied with their body image (difference between self and ideal body size = 0); women who felt too small regarding their ideal (difference between self and ideal <0) and women who felt that they were too large regarding their ideal (difference between self and ideal >0). Overweight or obese women before pregnancy who perceived their body size as too small were scarce and were not considered in this analysis.

### Follow-up Evaluation

Four years after the Generation XXI child’s birth, trained interviewers carried out face to face interviews regarding the health of the mother and the child and performed anthropometric evaluations of both. Weight was measured and recorded to the nearest 0.1 kg and height was measured without shoes to the nearest 0.1 cm. BMI at follow-up was categorized according to the same guidelines used for prepregnancy BMI. Duration of breastfeeding was recorded as the period of time that the child received maternal milk exclusively or together with complementary foods, in weeks.

### Statistical Analysis

Statistical analysis was performed using the statistical software Stata 11.0 (College Station, TX, 2005). Sample characteristics are presented as counts and proportions for all categorical variables and mean and standard deviation (SD) for normally distributed continuous variables. Proportions were compared using the chi-square test and continuous variables with ANOVA.

We defined BMI change as the difference between the BMI at follow-up and the prepregnancy BMI. To evaluate the association between BIS and BMI change over 4 years (continuous variable), linear regression models were used, taking women who were satisfied with their image as the reference class of exposure. Crude and adjusted coefficients were calculated with the respective 95% confidence intervals (95% CI). Multinomial logistic regression models were used to compute odds ratios (OR) and 95% CI for the association between BIS and BMI 4 years after delivery (categorical variable). In each baseline BMI stratum, the reference category of the outcome (BMI at 4 years) corresponded to no change in BMI category. There were no obese women before pregnancy with normal weight at 4 years. Separate models were built for women with normal body mass index (BMI), overweight and obese before pregnancy, considering the hypothesis of different effects of BIS on later BMI according to those classes (formally an interaction and not confounding issue). The final model in each pre-pregnancy BMI group was fitted to quantify the association between BIS (independent variable or exposure) on BMI at 4 years (dependent variable or outcome), adjusting for age at delivery, education, income, smoking before pregnancy, parity, weight gain during pregnancy and duration of breastfeeding, which were a priori considered potential confounders of the association, taking into account the literature review. The multivariable adjusted odds ratios are the final estimates considered to best represent the independent effect of the exposure on the outcome. Both the exposure and the outcome have more than 2 classes and the effects are expressed for each class in comparison with the reference.

## Results

The characteristics of the study participants are summarized in Table 1, according to women’s prepregnancy BMI. Overall, at birth, 35.2% of women were 25 to 29 years of age, 45.2% had less than 10 years of education and 34.7% had a household monthly income below 1000 euros. Before pregnancy, 26.0% of participants were overweight and 10.6% were obese. Women who were obese before pregnancy were older, had a lower educational level and a lower household monthly income than women with normal prepregnancy BMI. The proportion of women who smoked before pregnancy was lower among obese women than among normal and overweight women. Before pregnancy, overweight and obese women more frequently considered themselves too large regarding their ideal. Overweight women were also more likely to gain more weight than recommended during pregnancy and to present a lower increase in BMI over 4 years. Furthermore, obese women were less likely to breastfeed.

**Table 1:** Participants’ characteristics, according to prepregnancy BMI (n = 3612).

	Prepregnancy BMI				
	Total	Normal	Overweight	Obese	
	n (%)*	n (%)*	n (%)*	n (%)*	*p*
**Age (years)**					
<25	656 (18.2)	446 (19.5)	159 (16.9)	51 (13.3)	
25–29	1272 (35.2)	818 (35.7)	314 (33.4)	140 (36.6)	
30–34	1028 (28.5)	639 (27.9)	278 (29.6)	111 (29.0)	
≥35	656 (18.2)	386 (16.9)	189 (20.1)	81 (21.2)	0.015
**Education (years)**					
≤9	1633 (45.2)	885 (38.7)	496 (52.8)	252 (65.8)	
10–12	1028 (28.5)	664 (29.0)	283 (30.1)	81 (21.2)	
>12	951 (26.3)	740 (32.3)	161 (17.1)	50 (13.1)	<0.001
**Household income (€/month)**					
<1000	1253 (34.7)	709 (31.0)	356 (37.9)	188 (49.1)	
1001–1500	994 (27.5)	615 (26.9)	273 (29.0)	106 (27.7)	
≥1501	1058 (29.3)	758 (33.1)	229 (24.4)	71 (18.5)	
Does not know/Prefers not to answer	307 (8.5)	207 (9.0)	82 (8.7)	18 (4.7)	<0.001
**Smoking before pregnancy**					
Never smokers	2273 (62.9)	1411 (61.6)	594 (63.2)	268 (70.0)	
Current smokers	900 (24.9)	609 (26.6)	224 (23.8)	67 (17.5)	
Former smokers	439 (12.2)	269 (11.8)	122 (13.0)	48 (12.5)	0.003
**Body image satisfaction**					
Too small	256 (7.1)	238 (10.4)	16 (1.7)	2 (0.5)	
Satisfied	1553 (43.0)	1260 (55.1)	256 (27.2)	37 (9.7)	
Too large	1803 (49.9)	791 (34.6)	668 (71.1)	344 (89.8)	<0.001
**Parity**					
1	2092 (57.9)	1450 (63.4)	473 (50.3)	169 (44.1)	
2	1195 (33.1)	680 (29.7)	358 (38.1)	157 (41.0)	
≥3	325 (9.0)	159 (7.0)	109 (11.6)	57 (14.9)	<0.001
**Weight gain during pregnancy** a					
Below recommended	817 (22.6)	638 (27.9)	88 (9.4)	91 (23.8)	
As recommended	1294 (35.8)	911 (39.8)	289 (30.7)	94 (24.5)	
Above recommended	1501 (41.7)	740 (32.3)	563 (59.9)	198 (51.7)	<0.001
**BMI at follow-up (kg/m^2^)**					
18.5–24.9	1641 (45.4)	1563 (68.3)	78 (8.3)	0 (0.0)	
25.0–29.9	1178 (32.6)	654 (28.6)	487 (51.8)	37 (9.7)	
≥30	793 (22.0)	72 (3.2)	375 (39.9)	346 (90.3)	<0.001
**BMI change (kg/m^2^)** b					
Mean (SD)	2.02 (2.71)	1.85 (2.19)	2.40 (3.13)	2.10 (3.99)	<0.001
**Breastfeeding**					
Never	216 (6.0)	122 (5.3)	62 (6.6)	32 (8.4)	
≤26 weeks	2074 (57.4)	1306 (57.1)	537 (57.1)	231 (60.3)	
>26 weeks	1322 (36.6)	861 (37.6)	341 (36.3)	120 (31.3)	0.040

BMI, body mass index; SD, standard deviation.

a According to the Institute of Medicine classification (2009).

b Computed as the difference between BMI 4 years after delivery and BMI before pregnancy.

* except for BMI change, summarized as mean and standard deviation.

During the first 4 years after pregnancy, BMI increased, on average, 2.02 kg/m^2^. Four years after delivery, 28.6% of women with normal BMI before pregnancy were overweight, 39.9% of overweight women at baseline were obese and more than 90% of the obese women before pregnancy remained in the same BMI category (Table 1).

Among women who had normal prepregnancy BMI, those who felt too small regarding their ideal had a smaller increase in BMI (β = −0.25 kg/m^2^; 95%CI: −0.54 to 0.03) than women who were satisfied with their body image, after adjustment for confounders. In the same BMI category, those who felt too large had a significant additional increase of 0.41 kg/m^2^ (95%CI: 0.23 to 0.60) in BMI during the same period of time than women who were satisfied with their body image. In overweight women, a non-significant higher increase in BMI was observed (β = 0.12; 95%CI: −0.32 to 0.56) among too large women than in those who considered themselves satisfied. In obese women, those who felt too large had a non-significantly lower increase in BMI (β = −0.18; 95%CI: −1.49 to 1.12) (Table 2).

**Table 2:** Crude and adjusted mean difference (β) of BMI change from before to 4 years after pregnancy estimated by linear regression, according to body image satisfaction, in normal, overweight and obese women before pregnancy.

	BMI change (at 4 years – before pregnancy)			
	β (95%CI)			
	Mean (SD)	Crude	Adjusted*	
**Normal BMI before pregnancy**				
Too small	1.61 (2.04)	−0.05 (−0.35 to 0.25)	−0.25 (−0.54 to 0.03)	
Satisfied	1.66 (2.07)	0 (Ref)	0 (Ref)	
Too large	2.23 (2.38)	0.57 (0.38 to 0.76)	0.41 (0.23 to 0.60)	
**Overweight before pregnancy**				
Satisfied	2.36 (3.11)	0 (Ref)	0 (Ref)	
Too large	2.43 (3.14)	0.08 (−0.38 to 0.52)	0.12 (−0.32 to 0.56)	
**Obese before pregnancy**				
Satisfied	2.45 (3.01)	0 (Ref)	0 (Ref)	
Too large	2.06 (4.10)	−0.39 (−1.75 to 0.96)	−0.18 (−1.49 to 1.12)	

95%CI, 95% confidence interval; BMI, body mass index.

* Adjusted for age (<25; 25–29; 30–34; ≥35), education (≤9; 10–12; ≥12), household monthly income (<1000€; 1001–1500€; ≥1501; does not know/prefers not to answer), smoking before pregnancy (never, current, former), parity(1;2;≥3), weight gain during pregnancy (below recommended; as recommended; above recommended) and duration of breastfeeding (never; ≤26weeks;>26weeks).

Table 3 presents the association between BIS before pregnancy and BMI 4 years after delivery, by categories of prepregnancy BMI. After adjustment for the same variables, among women with normal BMI before pregnancy, those feeling that their body was too small regarding their ideal were significantly less likely to become overweight than to remain normal 4 years after delivery (OR = 0.63; 95%CI: 0.44–0.91), compared to those satisfied with their body image, and even less likely to become obese in the same period of time (OR = 0.21; 95%CI: 0.05–0.91). In the same BMI category, a significant opposite association was observed for women who felt that their body was too large regarding their ideal, with these women being more likely to become overweight and obese 4 years after delivery (OR = 2.12; 95%CI: 1.73–2.59 and OR = 3.42; 95%CI: 2.02–5.79, respectively) than those satisfied with their body image. Overweight women, and despite the lack of statistical significance, who felt too large regarding their ideal were 36% less likely to decrease in BMI category and 33% more likely to increase BMI during the same period of time. Among women obese before pregnancy, BIS was not associated with change in BMI class (Table 3).

**Table 3:** Adjusted odds ratio for the association between body image satisfaction before pregnancy and BMI 4 years after delivery estimated by multinomial logistic regression, stratified by prepregnancy BMI.

	BMI 4 years after delivery		
	OR (95%CI)*		
	Normal	Overweight	Obese
**Normal BMI before pregnancy**			
Too small	1	0.63 (0.44–0.91)	0.21 (0.05–0.91)
Satisfied	1	1	1
Too large	1	2.12 (1.73–2.59)	3.42 (2.02–5.79)
**Overweight before pregnancy**			
Satisfied	1	1	1
Too large	0.64 (0.38–1.08)	1	1.33 (0.96–1.84)
**Obese before pregnancy**			
Satisfied	−	1	1
Too large	−	1.76 (0.38–8.15)	1

95% CI, 95% confidence interval; BMI, body mass index; OR, odds ratio.

* Adjusted for age (<25; 25–29; 30–34; ≥35), education (≤9; 10–12; ≥12), household monthly income (<1000€; 1001–1500€; ≥1501; does not know/prefers not to answer), smoking before pregnancy (never, current, former), parity(1;2;≥3), weight gain during pregnancy (below recommended; as recommended; above recommended) and duration of breastfeeding (never; ≤26weeks;>26weeks).

## Discussion

In this sample of Portuguese mothers, the prevalence of overweight/obesity was high and weight increased considerably over 4 years after pregnancy, moving a large proportion of women upwards across BMI categories. The results of this study suggest that BIS before pregnancy was associated with weight change in the postpartum period and this association depended on the BMI before pregnancy.

Prevalence estimates of overweight/obesity in women before pregnancy vary across countries and the variability of methods in data collection among different studies increases the difficulty in making valid comparisons. A study conducted in the United States, using self-reported data, reported 22% of prepregnancy obesity in 2002–2003 [29]. Another study in the same country only with low-income women reported a prevalence of 28.3% of obesity before pregnancy in 2008, using measured height and self-reported prepregnancy weight [30]. Taking into account only measured data, a Portuguese study showed a female overweight and obesity prevalence of 34.4% and 13.4%, respectively, in 2003–2005 [3]. Relying on self-reported weight and height, the 2005/2006 Portuguese National Health Survey presented an overweight prevalence of 19.6% and obesity of 9.4% among women aged 18 to 44 years old [31].

The results of this study reveal that women were heavier 4 years after delivery than they were before pregnancy. We believe that it mainly resulted from an excessive weight gain during pregnancy which is retained after delivery. In fact, a temporal trends analysis of maternal prepregnancy body weight in Canada, showed that the critical age for weight gain before pregnancy is between 20 and 24 years [32], at the transition from adolescence to adulthood, when women gain independence in food choices and preparation. In our sample, the mean age of women was almost 30 years which is above the “critical age” threshold for spontaneous weight gain unrelated with pregnancy and childbirth.

Pregnancy causes direct changes in body size and weight, which have a high potential to influence BIS. In this context, most studies have focused on body image during pregnancy [33], [34] and not after childbirth. Within the ANC project, 2.5 years after delivery, excessive weight gain was associated with increased maternal dissatisfaction with body size: mothers who felt too large regarding their ideal immediately after pregnancy had significantly greater long term weight gains than women who had no dissatisfaction [22].

A relation between body size perception and change in BMI over 13 years has been recently reported, in a community sample of blacks and whites from the CARDIA study [35]. In this study, normal weight white women who were satisfied with their body size had a lower annual BMI change than did normal weight white women who perceived that they were a bit too large, suggesting that greater satisfaction with body size results in better weight control. A different pattern emerged among obese women, with those who perceived their body size as much too large gaining less weight than obese women who were more satisfied with their body size. Comparing to the current study, with almost exclusively white women, similar results were obtained except for the obese women for whom we found only a non-significant decrease in BMI. In this particular category, 80% of women considered themselves too large regarding their ideal silhouette and only 10% changed BMI category from overweight to obese. Therefore, our approach may have had low sensitivity to reach more solid conclusions for the obese women.

The longitudinal nature of the study allowed us to test the temporal relation between body image and BMI change. Also, the large number of participants allowed the control for many important confounders of this association. However, some limitations should be pointed. First, prepregnancy weight was self-reported and, because weight tends to be underreported by women [36], it may lead to an underestimation of the overweight and obesity prevalence, and consequently an overestimation of subsequent weight gain. However, self-reported prepregnancy body weights correlate very well with current measured body weights in women of reproductive age, although the size of the error varies according to ethnicity, socioeconomic position and prepregnancy body size, being larger among overweight women [26]. Nevertheless, the change in BMI category may be less subject to bias from reporting errors, compared with average postpartum weight change [26]. Therefore, self-reported prepregnancy weight provides reasonable estimates of BMI category since the reporting error (1–2 Kg) is a small percentage of total body weight. Also, when considering the analysis with BMI 4 years after delivery using self-reported weight (data not shown), the results were unchanged, supporting that the associations between BIS and BMI did not result mostly from the use of misreported prepregnancy weight and objectively measured weight at 4 years.

Regarding BIS, an important limitation of the study is that the silhouettes assessed at birth are representing prepregnancy body shapes and this retrospective characterization may reflect mothers’ tendency to idealize their prepregnant figure. Despite these limitations, Stunkard silhouettes have some advantages, allowing a uniform approach to study subjects in a not overly time-consuming procedure. The credibility of this method for scientific research is reinforced by the application of this tool in recent investigations [37]–[39]. Despite the methodological problems with the use of figural drawing scales, these are the most widely adopted measures of dissatisfaction with body size [40]. Even knowing that the Stunkard silhouettes may not be sensitive enough to pick up subtle differences in body size perception, [41], our results showed strong and significant effects with this measure, reinforcing the high impact of BIS on weight control.

Although this is clearly an incomplete view of the body image construct, the meaning of body image satisfaction is clear and relevant for non-specialists, and, from a public health perspective, we wanted to demonstrate that the evaluation of this dimension with this scale has the potential to identify women who are more susceptible of becoming overweight or obese and, therefore, to contribute to prevent the rising rates of obesity.

Since prenatal care is one of Portugal’s health care system most successful areas, with practically 100% coverage and adequate prenatal care in the vast majority of pregnancies, these stage of women’s life should be seen as an opportunity for prevention, not only for the children but also for their mothers. BIS could be an important factor to earlier identify women who are more susceptible of gaining weight during pregnancy and maintain it after delivery in order to optimize women’s wellness and subsequent pregnancy outcomes.

This scale is validated to the Caucasian population [42] and previous research in this cohort showed that only 3% of the cohort was not Portuguese or, at least, not European [43].

Furthermore, despite our large sample size, there were few obese women before pregnancy who felt satisfied with their own body image (9.7%). Therefore, the estimates are more imprecise for this group of women.

Despite the high proportion of participation at follow-up, mothers who attended the follow-up re-evaluation were older, more educated, had a higher income and were more likely to felt too large regarding their ideal before pregnancy. Since higher socioeconomic status is associated with lower BMI [44], we may be underestimating overweight and obesity 4 years after delivery and, consequently, the associations that were found could be stronger.

Maternal body weight increases with parity [45]. Therefore, to eliminate the possible effect of weight gain due to previous pregnancies, we performed a stratified analysis by parity and no differences were found between primiparous and multiparous women (data not shown).

One needs to address possible contributing mechanisms to explain the reported association. Low body image perceptions are associated with higher incidences of depression [46], mostly due to the discrepancy between current beauty ideals and body size of real women [47]. However, depression is a very complex phenomenon and it is not clear if it is a cause or a consequence of body image dissatisfaction, since the onset of both conditions is difficult to define precisely in time. A diagnosis of depression before pregnancy (ever) or during the 4 years after delivery were not associated with BIS or BMI in this cohort (data not shown), but these are likely to be insensitive to capture the mood changes within the spectrum of non-pathological mental health that would be relevant in this causal pathway.

In conclusion, our results suggest that satisfaction with body size plays an independent role in maternal body weight 4 years after the birth of a child, not only in women who already belonged to a high risk category, but also for those with a normal BMI who would not be considered a vulnerable group a priori. This apparently “healthy” group may be masked before pregnancy but also constitutes an important target for public health interventions. These findings support the importance of working on expectations and motivations to attain a goal, since more realistic body size goals can make women less susceptible of gaining weight during pregnancy and maintaining it after delivery.
